# A systematic, standards-based, participatory assessment of a continuous quality improvement project in Kyrgyzstan and Tajikistan: results for neonatal care

**DOI:** 10.7189/jogh.15.04162

**Published:** 2025-05-05

**Authors:** Khatuna Lomauri, Tatiana Caraus, Irina Stepanova, Sagynbu Abduvalieva, Firuza Zakirova, Shoira Yusupova, Nurshaim Tilenbaeva, Oleg Kuzmenko, Martin W Weber, Sophie Jullien, Kyrgyzstan-Tajikistan Quality of Hospital Care for Mothers and Children study group, Dmitrii Beglitse, Tinatin Gagua, Ana Calancea, Olga Teplyakova, Ksenia Gorina, Ksenia Gerasimova, Eleonora Zhumalieva, Chinara Abdyrahmanova, Minura Mamarasul Kyzy, Isaeva Mavjuda, Muzaffarrov Shamshod, Meniqulov Anvar

**Affiliations:** 1 Department of Neonatology, Tbilisi State Medical University, Tbilisi, Georgia; 2 Department of Science, Innovation and Research, Institute for Mother’ and Child, Chisinau, Republic of Moldova; 3 State Clinical Hospital named after S. Grinberg, Perm, Russian Federation; ^4^Ministry of Health of the Kyrgyz Republic, Bishkek, Kyrgyzstan; 5 NICU of the National Scientific and Research Centre for Obstetrics, Gynecology And Perinatology of the Republic of Tajikistan, Dushanbe, Tajikistan; 6 WHO Country Office of Tajikistan, Dushanbe, Tajikistan; 7 WHO Athens Office for quality of Care and Patient Safety, Athens, Greece; 8 WHO Regional Office for Europe, Division of Country Health Policies and Systems, Policy and Governance in Health Unit, Copenhagen, Denmark

## Abstract

**Background:**

Progress in reducing neonatal mortality in low- and middle-income countries in Central Asia, such as Kyrgyzstan and Tajikistan, has been slow. We aimed to explore deficiencies in neonatal care quality, highlight areas for improvement, and propose evidence-based actions to promote further progress in both countries.

**Methods:**

In this mixed-methods study, we evaluated neonatal care quality before and after a two-year quality improvement (QI) project in nine maternity hospitals in Kyrgyzstan and ten in Tajikistan. Through service visits, medical record reviews, direct observations, and interviews with staff and mothers, a multidisciplinary team of national and international experts and local managers assessed care quality using a tool developed by the World Health Organization, with scores ranging from 0 to 3. The QI cycle included establishing a QI team, developing an action plan, building healthcare providers’ capacity, providing supportive supervision, and initiating policy changes, with all initiatives reviewed during semi-annual collaborative QI meetings.

**Results:**

At baseline, neonatal care quality was suboptimal (scores 1.0–1.9) across all assessed areas. By the project’s conclusion, improvements were seen in routine care (Kyrgyzstan +0.4, Tajikistan +0.5), sick newborn care (+0.6 in both), hospital care accessibility (Kyrgyzstan +0.5, Tajikistan +0.8), and monitoring/follow-up (Kyrgyzstan +0.3, Tajikistan +0.9). Tajikistan also progressed in guidelines, training, audits (+0.8), and maternal/newborn rights (+0.5). However, hospital support services remained below good practice standards (<2.0) in both countries, and no significant improvements (<0.3) occurred in advanced newborn care or infection prevention.

**Conclusions:**

Comprehensive QI interventions have led to significant enhancements in neonatal care quality in both Kyrgyzstan and Tajikistan. However, critical gaps persist in certain essential areas that must be addressed. Ongoing, evidence-based QI efforts, alongside close monitoring, nationwide expansion, and strong government support, are essential to guarantee continued progress in these countries.

Neonatal deaths declined from 5.0 million in 1990 to 2.3 million in 2022 worldwide. Yet, nearly half (47%) of all deaths in children under five occurred during the neonatal period, *i.e.* the first 28 days [1]. Most of these deaths happen at birth or within the first 24 hours [2,3]. For this reason, improving care quality and implementing evidence-based practices remain key strategies for reducing neonatal mortality and improving newborn outcomes [4–6]. Care quality assessments help identify and address service gaps, as evidenced in Uzbekistan, where a standard-based participatory assessment led to improvements in neonatal care [7]. However, challenges still persist, including the absence of standards, workforce shortages, limited continuing education, and inconsistent support. Delivering life-saving interventions in emergency newborn care facilities is vital, especially given the slow progress in reducing neonatal deaths [8].

Quality improvement (QI) efforts in low- and middle-income countries (LMICs) have shown promising results, such as enhancing newborn care in Somalia [9], midwifery capacity in Papua New Guinea [10], and medical care in Zambia [11]. Still, sustaining improvements at central and district levels remains difficult due to limited public sector capacity. In Zimbabwe, continuous QI efforts have improved postnatal and maternal care [12], although the impact has been limited by fragmented quality assurance policies, staff shortages, and gaps in QI training. A neonatal-specific antimicrobial stewardship programme established in Lebanese neonatal intensive care units (NICUs) and employing a multidisciplinary approach, incorporating algorithms, audit, and feedback, has reduced antimicrobial use [13]. Yet, its sustainability has been challenged by the economic crisis straining the country’s healthcare system.

While QI efforts are expanding in LMICs, comprehensive outcome evaluations remain scarce [14]. Key challenges include identifying context-appropriate interventions to enhance the quality of neonatal care and ensuring their sustainability.

The quality of care for neonates is particularly pressing in Central Asia’s LMICs, such as Kyrgyzstan and Tajikistan, which were the focus of this study. Although the health sector has seen some advances in neonatal outcomes over the past decade, the pace of progress is insufficient to achieve the Sustainable Development Goals (SDGs) milestones, with neonatal mortality rates remaining unacceptably high in both countries, at 11.8 in Kyrgyzstan and 13.1 in Tajikistan in 2022 [15].

The primary objective of this study was to systematically assess the quality of newborn care in Kyrgyzstan and Tajikistan, with an aim to explore deficiencies in neonatal care, highlight areas for improvement, and propose evidence-based actions to promote further progress in both countries. This analysis specifically focusses on neonatal health, while details of the assessment process, other aspects of maternal and child care, and country-specific elements are currently under review in a separate manuscript and will be reported elsewhere (manuscripts in review).

## METHODS

A pre-post mixed methods study implemented in Kyrgyzstan and Tajikistan aimed to improve hospital care for mothers and children, thereby reducing maternal and child mortality in line with SDG targets. Supported by World Health Organization (WHO) Regional Office for Europe and the countries’ Ministries of Health (MoH), we conducted a baseline assessment in October 2021, with project progress evaluated in November 2023 using the same tool.

### Sampling

The MoHs of Kyrgyzstan and Tajikistan, in collaboration with WHO regional offices, selected nine and ten pilot district hospitals, respectively, based on geographic distribution, ensuring representation from three regions/provinces and levels of care, including at least one referral hospital.

### Assessment tool

We used the WHO Hospital Care for Mothers and Newborns Quality Assessment Tool (2014, updated 2021) [16] which evaluates care from admission to discharge across three main areas: hospital support services, case management, and policy/service organisation. Case management aligned with the WHO Europe’s Effective Perinatal Care (EPC) standards [17]. We collected data from hospital statistics, medical records, direct observation, and staff and mother interviews, while we assessed mothers’ satisfaction and existing barriers through anonymous interviews.

We scored each item using the following system, whereby each block included sub-blocks with multiple contributing indicators:

- 0: Very low or absent quality of care, posing systematic and serious risks to patient health.
- 1: Inadequate care, with significant risks and serious violations of women’s and newborns’ rights.
- 2: Insufficient care not meeting standards, but without direct health hazards; rights mostly respected.
- 3: Care met international standards, with no or minimal changes needed.

### Assessment team

An international assessment team comprised experienced professionals from key disciplines, including obstetrics, midwifery, and neonatology, all with extensive experience as WHO EPC trainers. Each country's MoH also selected a national team based on criteria prioritising a multidisciplinary approach and relevant experience. The national teams were larger to help build local capacity for conducting assessments.

### Assessment process

A five-day workshop trained the national team on the tool, scoring system, and EPC standards. Scores were calculated as the mean of assessed items and were further enriched through interviews with health workers and patients by capturing perceptions of care quality.

Findings and recommendations were shared at facility feedback meetings, where leadership helped develop action plans. This participatory approach was welcomed by professionals accustomed to bureaucratic oversight. A detailed report and action plan were submitted to the MoH and partners and discussed at a national workshop. To ensure consistency in methods and scoring, the same international teams conducted both baseline and end-line assessments in each country.

### Key QI activities

We developed a 24-month action plan for each hospital, outlining priorities, responsibilities, and timelines, with key activities including:

- Establishing QI teams and hospital-specific improvement plans.
- Building capacity for supportive supervision and guiding visits.
- Providing technical support for creating, updating, and implementing guidelines.
- Training healthcare providers in clinical knowledge and skills (EPC training).
- Developing or updating local protocols.
- Holding semi-annual meetings to review progress, address barriers, and plan monitoring visits.

### Data analysis

We used a Wilcoxon signed rank test to analyse changes in the various domains of neonatal care from baseline to post-intervention, with a *P*-value <0.05 indicating statistical significance. We conducted the analysis in SPSS, version 27 (IBM Corp., Armonk, New York, USA).

### Ethics approval

The MoH in each country approved the study (Kyrgyzstan: orders #1392 (4 October 2021) and #1218 (12 October 2023), Tajikistan: orders #993 (09 November 2021) and #688 (26 October 2023)). Hospital management approved the tools and methods and all participants provided informed consent.

## RESULTS

### Hospital support services

**Figure 1:**
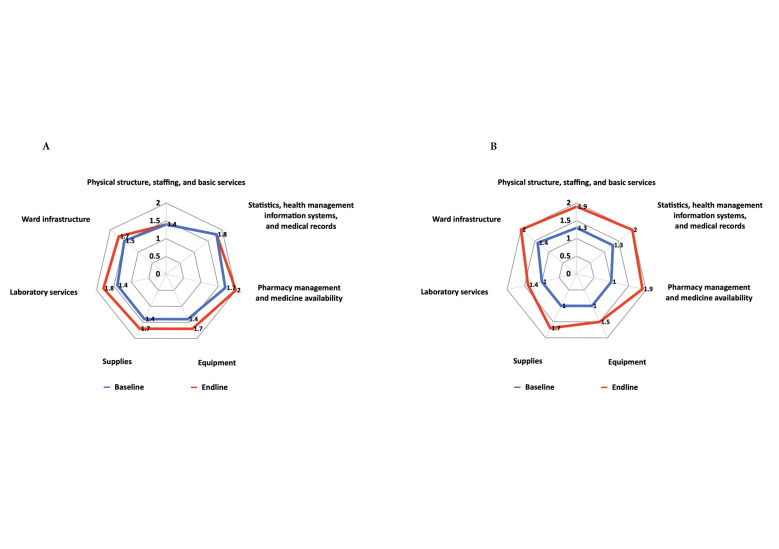
Comparison of changes in hospital support services. **Panel A.** Kyrgyzstan**. Panel B.** Tajikistan**.**

The mean score across all variables in Kyrgyzstan improved slightly, from 1.5 at baseline to 1.7 at the end-line assessment (*P* = 0.214). Positive changes, though non-significant, were detected in pharmacy management and medicine availability, laboratory services, equipment, and supplies (**Figure 1**, Panel A). In Tajikistan, the baseline mean score for this section was 1.2, compared to 1.8 at the end-line assessment (*P* < 0.001). Significant improvements were observed in all sub-blocks: physical structure, staffing, and basic services (*P* = 0.011), health management information systems and medical records (*P* = 0.005), pharmacy management and medicine availability (*P* = 0.005), equipment (*P* = 0.008), supplies (*P* = 0.011), laboratory services (*P* = 0.049), and ward infrastructure (*P* = 0.008) (**Figure 1**, Panel B). Mean scores across all variables in hospital support services in both countries remained below the good practice standards (<2.0).

### Case management

#### Routine newborn care

**Figure 2:**
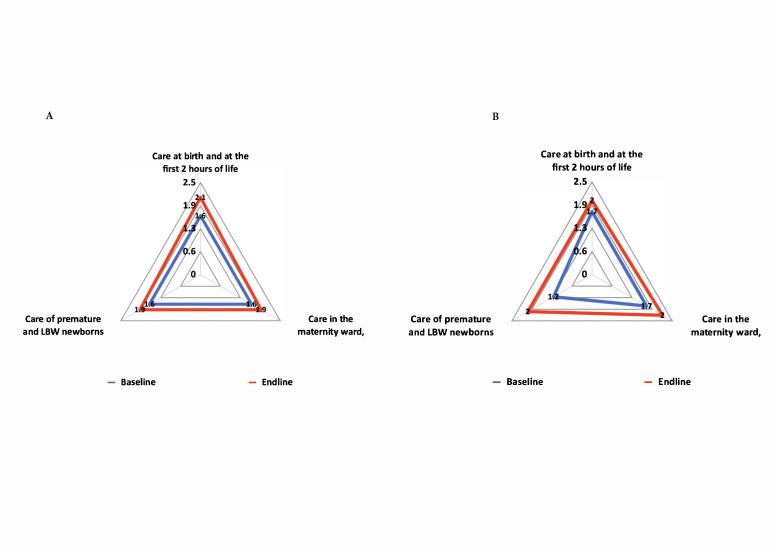
Comparison of changes in routine newborn care**.** **Panel A.** Kyrgyzstan**. Panel B.** Tajikistan**.**

Routine newborn care in Kyrgyzstan improved significantly (*P* = 0.047), with a mean score increasing from 1.6 to 2.0 at the end-line. Significant improvements were noted in care at birth and during the first two hours of life (*P* = 0.016), alongside improvements, though non-significant, in maternity ward care (*P* = 0.088) and the care of premature and low birth weight (LBW) infants (*P* = 0.084) (**Figure 2**, Panel A).

In Tajikistan, routine newborn care improved significantly (*P* = 0.007), with an end-line mean score reaching 2.08, up from 1.6 at baseline. Enhancements were noted across all variables: care at birth and in the first two hours (*P* = 0.016), care in the maternity ward (*P* = 0.033), and care for premature and LBW infants (*P* = 0.005) (**Figure 2**, Panel B).

#### Sick newborn care

At baseline, the mean score in Kyrgyzstan for this section across all variables was 1.2, indicating a significant increase to 1.8 at the end-line assessment (*P* = 0.02). Improvement in care for specific conditions was even more notable (*P* = 0.021).

In Tajikistan, the baseline mean score for sick newborn care was 1.3, compared to the end-line score of 1.9 (*P* = 0.005). We identified a significant improvement in the sub-block of care for specific conditions (*P* = 0.007) and non-significant improvement in general care (*P* = 0.114).

#### Advanced newborn care

**Figure 3:**
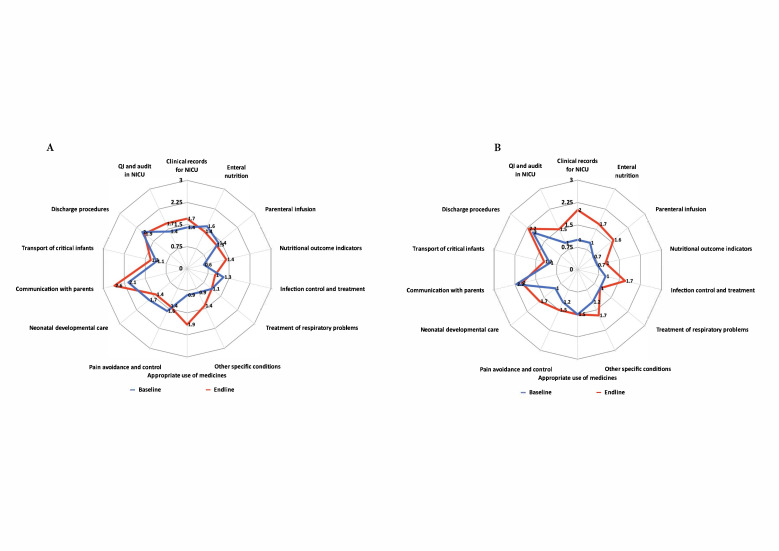
Comparison of changes in advance newborn care. **Panel A.** Kyrgyzstan**. Panel B.** Tajikistan**.** NICU – neonatal intensive care unit.

In Kyrgyzstan, the baseline mean score across all variables for advanced newborn care was 1.4, increasing, though non-significantly (*P* = 0.31), to 1.6 at the end-line assessment. We detected positive changes in the following variables: medical records for NICUs, parenteral infusion, nutritional outcome indicators, and communication with parents; however, only the improvement in appropriate use of medicines reached statistical significance (*P* = 0.038) (**Figure 3**, Panel A).

In Tajikistan, the baseline mean score across all variables remained unchanged at 1.6 during the end-line assessments (*P* = 0.144). We saw positive, though non-significant changes in all variables, with clinical records for NICU being the only exception (*P* = 0.046) (**Figure 3**, Panel B).

### Monitoring and follow-up

The baseline mean score in Kyrgyzstan for this section was 1.7, with a significant increase to 2.0 (*P* = 0.0083) at the end-line assessment. In Tajikistan, the mean score for monitoring and follow-up significantly increased from 1.5 at baseline to 2.35 (*P* = 0.005) at the end-line assessment.

### Policies and organisation of care

Concerning the policies and organisation of services, the baseline mean scores for all sub-blocks (access to hospital care and continuity of care guidelines, training and audit, mothers' and newborns' rights, infection prevention) were below the good practice standards (<2.0) in both Kyrgyzstan and Tajikistan.

#### Access to hospital care and continuity of care

In Kyrgyzstan, this section showed a significant improvement, with the baseline mean score increasing from 1.5 to 2.0 at the end-line assessment (*P* = 0.008). Similarly, the baseline mean score of 1.2 for this section in Tajikistan significantly increased to 2.0 at the end-line assessment (*P* = 0.005)**.**

#### Guidelines, training and audit

For this section, we noted a slight, non-significant, increase in the mean score from 1.5 to 1.7 (*P* = 0.295) in Kyrgyzstan. In contrast, the improvement in the mean score from 1.2 to 2.0 in Tajikistan was statistically significant (*P* = 0.005).

#### Mothers’ and newborns’ rights

In Kyrgyzstan, there was a minor and non-significant increase in the mean score from 1.6 to 1.7 (*P* = 0.182) across this section. In Tajikistan, we detected a significant change in the mean score from 1.3 to 1.8 (*P* = 0.014).

#### Infection prevention

In Kyrgyzstan, the observed trend for this section was positive, though with a non-significant increase in the mean score from 2.0 to 2.3. Except for standard precautions (*P* = 0.043), all other variables, including infection control policies, hospital support services, and hand washing, showed non-significant improvements. In Tajikistan, the mean score for this section also increased not-significantly from 1.4 to 1.8 (*P* = 0.058). As for the respective variables, only infection control policies (*P* = 0.023) and hand washing (*P* = 0.011) showed significant improvements.

## DISCUSSION

Here we assessed the effectiveness of a combination of QI activities on neonatal care in 19 pilot maternity hospitals in Kyrgyzstan and Tajikistan using a pre-post mixed methods design. Our research offers unique contributions, including the use of a validated WHO tool and a participatory approach involving local and international experts. Recognising the challenges seen in previous research studies, we prioritised developing a draft action plan with a timeline and responsibilities during the baseline assessment. The leveraged momentum from quality gap feedback to foster commitment and designate accountable individuals. The experience of utilising the same assessment tool has provided comprehensive and detailed insights into quality gaps across 25 countries in various healthcare settings [18–20]. Since no other interventions were implemented in the selected hospitals, we are confident that the changes described can be attributed to the project activities. The baseline assessment revealed substandard neonatal care across all areas, with similar findings in both countries, likely due their comparable health systems – a legacy of the Soviet Union. We observed several significant improvements at the end of the project, particularly in routine and sick newborn care and monitoring and follow-up. We also noted some positive trends in specific fields of policy and organisation of care and hospital support services.

The progress observed suggests that multi-component QI programmes can enhance care quality. Below we discuss a combination of methods contributing to improved newborn care, particularly in LMICs such as Kyrgyzstan and Tajikistan. These include establishing a quality culture, active participation and teamwork, capacity building for healthcare providers through facility-level training, supportive supervision visits, and developing clinical guidelines, alongside semi-annual collaborative QI meetings to assess progress, identify weaknesses, and plan policy changes.

Establishing a QI team and involving hospital leadership were crucial for driving improvements. Similar studies in Zambia [11] and Yemen [21] highlight the importance of engaging local stakeholders. Initial assessments revealed gaps in infrastructure, personnel, and supplies. Facilities with proactive leadership addressed these issues creatively, leading to renovated neonatal departments, improved access to oxygen, and better water and temperature control. High-risk delivery rooms were equipped, and 10 out of 19 institutions created new neonatal nurse positions. The provision of basic medicines (vitamin K, tetracycline, glucose, and antibacterial drugs) has improved, with costs being borne by the medical institutions and supported by hospital management and the MoH’s key personnel.

We observed some improvements in laboratory services due to the efforts of the QI committee and management. Nonetheless, the quality of these services in both republics still requires enhancement. Persistent challenges include delays in test results and a lack of microbiology laboratories. While hospital support services have improved in both countries, progress has been more notable in Tajikistan, which had lower baseline scores.

The focus on participation and teamwork among obstetricians, neonatologists, and midwives has proven successful, similar to experiences in Honduras, where QI teams enhanced neonatal care [22]. Comparable findings in Brazil showed that participants remained committed to addressing quality gaps after the intervention [23]. This suggests that a QI culture was, to some extent, established as a result of the intervention. We observed successful teamwork in routine newborn care, with enhanced practices in the delivery room and postpartum unit, including better temperature control, immediate skin-to-skin contact, early breastfeeding initiation, delayed cord clamping, eye prophylaxis, mother-baby joint transfer to the postpartum unit, vitamin K prophylaxis, and early discharge. However, challenges remain in newborn care after cesarean sections, particularly in terms of missed opportunities for skin-to-skin contact and early breastfeeding due to mother-baby separation in the first 24 hours.

The capacity-building of healthcare providers through facility-level training is essential for improving maternal and newborn health. In Congo, these efforts have been crucial in enhancing provider performance and health outcomes [24]. Tailored EPC components in LMICs have shown better outcomes in newborn care [25]. The EPC programme generally focusses on a multidisciplinary approach, strengthening the role of nurses and midwives in case management. Over 500 obstetrician-gynecologists, neonatologists, midwives, and neonatal nurses have been trained in each country. This approach has heightened staff awareness of neonatal disorders such as hyperbilirubinaemia [26] and hypo/hyperglycaemia [27], and so on. The dissemination of evidence-based recommendations, such as the WHO charts for hyperbilirubinaemia management, has standardised clinical practices. The systematic identification of high-risk newborns has improved early detection and preventive measures for hypoglycaemia, including early enteral feeding for LBW infants.

Expanding clinical tasks for nurses and offering additional training opportunities have positively impacted patient monitoring in targeted facilities. However, critical gaps remain, including improper recording of relevant monitoring parameters and the lack of a link between nurse records and medical records.

Supportive supervision is a useful QI approach for clinical settings, challenging traditional notions of supervision by emphasising facilitation over inspection. This method promotes collaboration among local leadership to enhance clinical competencies and the performance of health workers in maternity settings. Supportive supervision has been implemented in various countries with different outcomes. In Zambia, QI activities initiated by Central Board of Health Leaders did not yield visible progress [11]. Conversely, a study from Zimbabwe highlighted supportive supervision as crucial to project success, though staff shortages and high turnover rates diminished its impact [12].

Within the scope of our project, supportive supervision and on-the-job training were identified as effective strategies for enhancing advanced newborn care in collaboration with local experts. This approach involved training of current supervisors and on-site capacity building supported by an international consultant. We observed some improvements in areas such as enteral nutrition, communication with parents, records for the NICU, and parenteral infusion, yet we detected no changes in respiratory and circulatory management, which are basic for managing critically ill patients. The limited skills of local experts, gaps in central-level supervision and district-level coaching, constrained budgets, and weak coordination may explain the lack of tangible progress in this critical area. Strengthening and sustaining supportive supervision is essential to ensure continuous improvement in newborn care. A study in Kyrgyzstan indicated a positive impact of this approach on paediatric care, highlighting the potential of focused support to replace conventional supervision [28].

The capacity-building of health workers to improve the quality of care, knowledge, adherence to clinical protocols, and clinical practice behaviour has been adopted in six South Asian countries as a QI approach to enhance neonatal health services [29]. Most facilities had neonatal protocols in place, with updates in Tajikistan contributing to improved outcomes. Healthcare providers have become more optimistic about the role of protocols in enhancing care. However, challenges remain in integrating recommendations into daily practice, especially in advanced newborn care, due to limited awareness and access to evidence, which the QI team must address.

Policy and procedural changes are essential for enhancing health system performance and establishing evidence-based practices, as shown in Papua New Guinea [10]. Semi-annual collaborative quality improvement meetings, held in close collaboration with the WHO Regional Office for Europe, the MoH, and local QI teams, were an integral part of the QI initiative within both countries' policy frameworks.

Although the project activities led to positive policy changes, the end-line assessments revealed persistent gaps due to fragmented and under-resourced QI efforts. Access to hospital care improved significantly in both republics, with newborns receiving 24/7 support. Facility managers displayed a better awareness of the mothers' and newborns' rights, while improving written and oral information for women and promoting mothers’ participation in neonatal care, especially for sick newborns. Nonetheless, challenges remain in budgeting, particularly concerning extra costs for drugs, supplies, and laboratory tests. In both countries, there are no standard operating procedures for transferring critically ill newborns to higher levels of care, affecting the rights of mothers and newborns. Similar findings have been reported in various health systems, including those in Albania, Turkmenistan, and Kazakhstan [30].

Infection prevention remains a concern for policy, despite advancements in hand hygiene and infection control in Tajikistan, alongside standard precautions in Kyrgyzstan. However, the absence of a nosocomial infection surveillance system continues to pose a challenge. Inadequate infrastructure, limited supplies, and insufficient staffing further impede infection prevention efforts. Similar findings have been reported across eight LMICs [31].

A criterion-based audit is a QI process aimed at improving patient care [32]. In Fiji, perinatal mortality audits have helped identify gaps in newborn care [33]. Despite the existence of case reviews and perinatal audits in Kyrgyzstan and Tajikistan, they often fostered a punitive culture, leading to missed opportunities for improvement. International expert involvement has proven valuable in shifting the focus from sanctions to identifying modifiable factors. Sustained training and capacity-building at the national level are essential for improving and maintaining newborn care quality.

### Limitations and strengths

This study has several limitations. The absence of a comparison group makes it difficult to attribute improvements in neonatal care solely to the QI intervention. An independent team collected and reported selected indicators in the intervention hospitals; further details will be reported elsewhere (manuscript under review)

While a standardised scoring system and consistent evaluation team enhanced reliability, full comparability across facilities was not guaranteed. However, assessor training, consistent team composition, score cross-checking, and international supervision likely mitigated this limitation.

The study also has several strengths, including the application of similar methodologies in both countries, the use of a validated assessment tool, the involvement of an external, qualified international committee in the evaluation process, and a systematic approach that engaged both the QI team and hospital managers. Furthermore, hospitals were selected to ensure representation from several regions/provinces and different levels of care, making the findings generalizable across both countries. These factors collectively enhance our understanding of the existing gaps and areas for improvement in neonatal care quality.

## CONCLUSIONS

A systematic, participatory approach, with targeted activities in Kyrgyzstan and Tajikistan, has shown improvement in neonatal quality care, as assessed using the WHO tool. This comprehensive framework was well-received by key staff and managers, fostering a quality ‘culture’. This study demonstrates that QI activities, such as promoting teamwork, providing on-the-job training, supportive supervision, developing and implementing protocols, and conducting semi-annual assessments, can enhance newborn outcomes in resource-constrained settings. This approach can optimise the use of limited government resources and support global initiatives towards shared goals.

While the project offered valuable lessons, it highlighted limitations in sustaining and expanding the QI process, largely due to a shortage of skilled experts, trainers, and financial resources. To maintain momentum and scale activities, the QI team’s motivation must be supported through enhanced teamwork, informed decision-making, and strong national leadership. Investments in governance, infrastructure, and human resources, alongside participatory quality improvement collaboratives, are essential to foster commitment to change.
